# AST/ALT ratio is an independent risk factor for diabetic retinopathy: A cross-sectional study

**DOI:** 10.1097/MD.0000000000038583

**Published:** 2024-06-28

**Authors:** Jian Luo, Fang Yu, Haifeng Zhou, Xueyan Wu, Quan Zhou, Qin Liu, Shenglian Gan

**Affiliations:** a Department of Endocrinology, Changde Hospital, Xiangya School of Medicine, Central South University, Changde, China; b Department of Gastroenterology, Changde Hospital, Xiangya School of Medicine, Central South University, Changde, China; c Department of Science and Education, Changde Hospital, Xiangya School of Medicine, Central South University, Changde, China.

**Keywords:** AST/ALT ratio, diabetic retinopathy, inflammation, oxidative stress

## Abstract

The aspartate to alanine transaminase (AST/ALT) ratio indicates oxidative stress and inflammatory reactions related to the occurrence of diabetic retinopathy (DR). Currently, there are no reports on the correlation between AST/ALT ratio and DR. Hence, this study aimed to explore the relationship between AST/ALT ratio and DR. This cross-sectional study utilized data from the Metabolic Management Center of the First People’s Hospital in City. In total, 1365 patients with type 2 diabetes mellitus (T2DM) participated in the study, including 244 patients with DR and 1121 patients without DR. We collected the results of fundus photography, liver function, and other research data and grouped them according to tertiles of AST/ALT ratios. DR prevalence was the highest in the group with the highest AST/ALT ratio (22.12%, *P *= .004). Both univariate (OR = 2.25, 95% CI: 1.51–3.34, *P *< .001) and multivariable logistic regression analyses (adjusted for confounding factors) showed that the risk of DR increased by 36% when the AST/ALT ratio increased by 1 standard deviation (SD) (OR = 1.36, 95% CI: 1.16–1.59, *P < *.001), and 29.3% was mediated by the duration of diabetes. A sensitivity analysis confirmed the stability of the results. This study showed that an increase in AST/ALT ratio is an independent risk factor for DR.

## 1. Introduction

Aspartate transaminase (AST) and alanine transaminase (ALT) are indicators of liver function. The AST/ALT ratio was first described by Fernando De Ritis in 1957.^[1]^ An increase in the AST/ALT ratio indicated oxidative stress and systemic inflammatory reaction.^[2]^

Diabetes is a chronic disease that adversely affects human health. The global prevalence of diabetes among people aged 20 to 79 years in 2021 is approximately 10.5% (5.366 million). Furthermore, it is estimated that the prevalence rate will increase to 12.2% (783.2 million) by 2045. China has the largest number of patients with diabetes globally, accounting for approximately 24% of the patients with diabetes worldwide.^[3]^ Diabetic retinopathy (DR) is one of the most common microvascular complications in patients with diabetes and has become a major cause of adult blindness.^[4]^ The global prevalence of DR among patients with diabetes was 22.27%. Further, approximately 103.12 million adults have DR, which is expected to increase to 160.5 million adults by 2045.^[5]^ Furthermore, the prevalence rate of DR in China is approximately 23%, which is increasing further, leading to a severe socio-economic burden.^[6]^ Early diagnosis and treatment of DR are crucial in diabetes; however, because early DR lacks clinical manifestations, it often remains undetected. Therefore, early identification of DR risk factors is necessary.

The AST/ALT ratio has been introduced as a novel metabolic, oxidative stress, and inflammatory marker,^[7,8]^ and is an indicator that is easily obtainable. Conversely, Type 2 diabetes mellitus (T2DM) is associated with inflammatory burden and oxidative stress. Inflammation and oxidative stress are implicated not only in T2DM but also in chronic microvascular complications including DR.^[9,10]^ We hypothesized that the AST/ALT ratio is a risk factor for DR. To the best of our knowledge, there are currently no reports on the correlation between the AST/ALT ratio and DR. Therefore, studying the AST/ALT ratio in relation to DR is warranted. This study was conducted on patients enrolled in the Metabolic Management Center (MMC) to ascertain the correlation between the AST/ALT ratio and DR.

## 2. Materials and methods

### 2.1. Study design and participants

**Figure 1.**
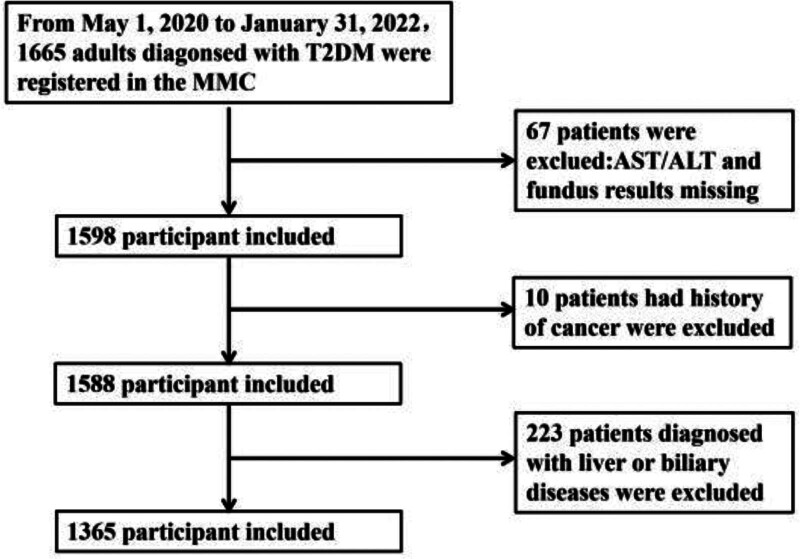
Flowchart of participants selected from the study. MMC = Metabolic Management Center, T2DM = Type 2 diabetes mellitus.

This study utilized a cross-sectional design, with the AST/ALT ratio as the independent variable and DR as the dependent variable. The participants comprised 1665 adults who had been diagnosed with type 2 diabetes mellitus (T2DM) and enrolled at the MMC of Changde Hospital, Xiangya School of Medicine, Central South University, between May 1, 2020 and January 31, 2022. The exclusion criteria for this study were as follows: lack of fundus photography and AST/ALT ratio results, liver conditions (such as fatty liver or viral hepatitis) or biliary tract disorders, and a history of cancer or hypertensive retinal diseases. Ultimately, 1365 participants were included in the data analysis (Fig. 1).

### 2.2. Data collection

Demographic information, including age, sex, smoking status, drinking habits, and duration of diabetes, was collected. Physical examination data included systolic blood pressure (SBP), diastolic blood pressure (DBP), and body mass index (BMI). Routine laboratory tests included glycated hemoglobin (HbA1c), fasting plasma glucose (FPG), 2-hour post-load glucose (2HPG), total bilirubin (TBIL), direct bilirubin (DBIL), triglycerides (TG), total cholesterol (TC), high-density lipoprotein cholesterol (HDL-C), low-density lipoprotein cholesterol (LDL-C), white blood cell (WBC) count, and fundus photography results. Non-mydriatic fundus images were captured using a fundus camera (Horus, DEC200) to acquire 45° color images centered on the optic papillae and macula of both eyes. Subsequently, the images were reviewed by the same ophthalmologist and categorized into DR and non-DR groups based on the established diagnostic criteria.^[11]^

### 2.3. Statistical analysis

Continuous variables are reported as either mean ± standard deviation or median and interquartile ranges (Q1–Q3). Classification variables are presented as frequencies or percentages. One-way ANOVA (for variables with a normal distribution), Kruskal–Wallis *H* test (for variables with a skewed distribution), and chi-square test (for categorical variables) were used to assess the differences among the AST/ALT ratio groups (tertiles). Univariate and multivariate logistic regression analyses were conducted to investigate the association between the AST/ALT ratio and DR. Since the AST/ALT ratio followed a normal distribution (0.96 ± 0.33), multiple logistic regression analyses were performed after converting the AST/ALT ratio to *Z* scores to improve result interpretation.^[12]^ A smooth curve fitting analysis was employed to explore the potential nonlinear relationship between the AST/ALT ratio and DR. Additionally, a sensitivity analysis was performed to ensure the robustness of the data analysis. Hierarchical and interactive analyses were utilized to explore the relationship between the AST/ALT ratio and DR in different subpopulations, with the *E* value used to assess potential unmeasured confounding factors.^[13]^ Notably, the *E* value was calculated as *E* value = OR+$\sqrt{OR\;\times \;OR\text{-}1}$.^[14]^ To detect potential mediation effects in the correlation between AST/ALT ratio and DR, a bootstrap test was performed. All statistical analyses were conducted using R statistical software packages and Empower Stats 4.1 (http://www.empowerstats.com, X&Y Solutions, Inc., Boston, MA, USA). Statistical significance was set at a 2-sided *P* value < .05 (two-sided) was considered statistically significant.

### 2.4. Ethics approval

The study protocol was approved by the Ethics Committee of Changde Hospital, Xiangya School of Medicine, Central South University (Approval No. YX-2023-016-01). All patients provided written informed consent prior to enrollment and the study was conducted in accordance with the principles outlined in the Declaration of Helsinki (1975).

## 3. Results

### 3.1. Baseline characteristics of all participants

A total of 1365 participants were included in this study, with an average age of 52.07 ± 11.15 years. In addition, 58.45% of the patients were male. Patients were divided into 3 groups based on the tertiles of the AST/ALT ratio. In comparison with the lowest AST/ALT ratio group (Q1), all variables were statistically significant, except for SBP. As the AST/ALT ratio increased, the proportion of DR also increased, with proportions in the 3 groups being 13.56%, 18.24%, and 22.12%, respectively (*P* = .004) (Table 1).

**Table 1:** General characteristics of participants by AST/ALT ratio tertile (N = 1365).

AST/ALT	Q1 (0.43–0.78)	Q2 (0.78–1.04)	Q3 (1.04–2.26)	*P* value	*P* value*
N = 1365	451	457	457		
Age (yrs)	48.23 ± 11.17	52.24 ± 10.17	56.30 ± 10.23	<.001	<.001
Sex					
Male	327 (72.51%)	268 (58.64%)	200 (43.76%)	<.001	–
Female	124 (27.49%)	189 (41.36%)	257 (56.24%)		
Smoking					
Never	247 (54.77%)	281 (61.49%)	327 (71.55%)	<.001	–
Ever	40 (8.87%)	36 (7.88%)	31 (6.78%)		
Everyday	164 (36.36%)	140 (30.63%)	99 (21.66%)		
Alcohol					
Never	282 (62.53%)	317 (69.52%)	343 (75.05%)	.001	–
Ever	39 (8.65%)	32 (7.02%)	21 (4.60%)		
Everyday	130 (28.82%)	107 (23.46%)	93 (20.35%)		
DM course (mo)	37 (9–81)	48 (17–100)	61 (20–122)	<.001	<.001
DR					
NDR	389 (86.44%)	372 (81.76%)	352 (77.88%)	.004	–
DR	61 (13.56%)	83 (18.24%)	100 (22.12%)		
DBP (mm Hg)	86.43 ± 11.56	82.44 ± 11.04	81.18 ± 10.85	<.001	<.001
SBP (mm Hg)	136.29 ± 18.35	134.34 ± 19.25	136.89 ± 21.16	.124	.235
BMI (kg/m^2^)	26.81 ± 3.68	25.60 ± 3.23	24.25 ± 3.07	<.001	<.001
WBC (*10^9/L)	6.48 ± 2.36	6.35 ± 1.75	6.17 ± 2.39	.096	.015
HBAIC (%)	8.66 ± 2.17	8.44 ± 2.29	7.83 ± 2.06	<.001	<.001
FPG (mmol/L)	9.41 ± 3.82	8.69 ± 3.23	8.01 ± 2.90	<.001	<.001
2HPG (mmol/L)	14.44 ± 5.72	13.40 ± 5.44	12.71 ± 4.98	<.001	<.001
TBIL (μmol/L)	13.19 ± 4.75	14.23 ± 6.95	13.05 ± 6.20	.006	.010
DBIL (μmol/L)	4.74 ± 1.93	4.88 ± 2.15	4.53 ± 2.21	.039	.004
UA (μmol/L)	345.58 ± 86.92	333.02 ± 85.20	325.38 ± 93.52	.003	<.001
TG (mmol/L)	2.24 (1.50–3.47)	1.81 (1.29–2.60)	1.58 (1.12–2.36)	<.001	<.001
TC (mmol/L)	5.10 ± 1.33	4.92 ± 1.18	4.85 ± 1.28	.010	.007
HDL-C (mmol/L)	1.19 ± 0.28	1.27 ± 0.34	1.32 ± 0.35	<.001	<.001
LDL-C (mmol/L)	2.90 ± 0.92	2.84 ± 0.93	2.67 ± 0.90	<.001	<.001

2HPG = 2-hour post-load glucose, AST/ALT = aspartate to alanine transaminase, BMI = body mass index, DBIL = direct bilirubin, DBP = diastolic blood pressure, DM course = course of diabetes, FPG = fasting plasma glucose, HbA1c = glycated hemoglobin, HDL-C = high-density lipoprotein cholesterol, LDL-C = low-density lipoprotein cholesterol, SBP = systolic blood pressure, TBIL = total bilirubin, TC = total cholesterol, TG = triglyceride white blood cell diabetes retinopathy, NDR = no diabetes retinopathy.

* *P* value: The continuous variable of non-normal distribution is calculated by Kruskal–Wallis *H* test. The continuous variables were expressed as mean ± SD or median (quartile range), and the categorical variables were presented as percentage.

### 3.2. Univariate analysis of risk factors for DR

As shown in Table 2, univariate analysis indicated that the course of DM (OR = 1.01, 95% CI: 1.00–1.01, *P *< .001), SBP (OR = 1.01, 95% CI: 1.00–1.02, *P *= .015), FPG (OR = 1.05,95% CI: 1.02–1.09, *P *= .005), and AST/ALT ratio (OR = 2.25, 95% CI: 1.51–3.34, *P *< .001) were all positively related to the occurrence of DR. However, sex, age, DBP, BMI, HbA1c, FPG, 2HPG, TBIL, DBIL, TG, TC, HDL-C, LDL-C, and WBC were not related to the occurrence of DR.

**Table 2:** Univariate analysis of DR.

	Statistics	OR (95% CI)	*P* value
Sex			
Male	795 (58.45%)	1.0	
Female	570 (41.55%)	1.17 (0.89, 1.55)	.2538
Age (yrs)	52.07 ± 11.15	1.01 (0.99, 1.02)	.3243
Smoking			
Never	889 (62.47%)	1.0	1.0
Ever	111 (7.80%)	0.91 (0.54, 1.53)	.7152
Everyday	423 (29.73%)	0.88 (0.65, 1.19)	.4089
Alcohol			
Never	972 (68.35%)	1.0	1.0
Ever	97 (6.82%)	1.16 (0.69, 1.96)	.5639
Everyday	353 (24.82%)	0.86 (0.62, 1.19)	.3597
DM course (mo)	49 (15.00–101.50)	1.01 (1.00, 1.01)	<.0001
DBP (mm Hg)	83.24 ± 11.32	1.00 (0.99, 1.01)	.9955
SBP (mm Hg)	135.56 ± 19.62	1.01 (1.00, 1.02)	.0151
BMI (kg/m^2^)	25.58 ± 3.53	0.98 (0.94, 1.02)	.3047
HBAIC (%)	8.37 ± 2.23	1.06 (1.00, 1.12)	.0564
FPG (mmol/L)	8.73 ± 3.39	1.05 (1.02, 1.09)	.0052
2HPG (mmol/L)	13.56 ± 5.46	1.02 (0.99, 1.04)	.1492
TBIL (μmol/L)	13.51 ± 6.06	0.98 (0.95, 1.00)	.0703
DBIL (μmol/L)	4.74 ± 2.13	0.95 (0.89, 1.02)	.1748
UA (μmol/L)	334.52 ± 88.64	1.00 (1.00, 1.00)	.8397
TG (mmol/L)	1.86 (1.30–2.83)	0.99 (0.95, 1.03)	.6022
TC (mmol/L)	4.96 ± 1.27	1.04 (0.93, 1.15)	.5198
HDL-C (mmol/L)	1.26 ± 0.33	1.11 (0.74, 1.67)	.6090
LDL-C (mmol/L)	2.81 ± 0.93	0.95 (0.82, 1.11)	.5358
WBC (*10^9/L)	6.35 ± 2.17	0.99 (0.92, 1.05)	.6978
AST/ALT	0.96 ± 0.33	2.25 (1.51, 3.34)	<.0001

2HPG = 2-hour post-load glucose, AST/ALT = aspartate to alanine transaminase, BMI = body mass index, DBIL = direct bilirubin, DBP = diastolic blood pressure, DM Course = course of diabetes, DR = diabetes retinopathy, FPG = fasting plasma glucose, HbA1c = glycated hemoglobin, HDL-C = high-density lipoprotein cholesterol, LDL-C = low-density lipoprotein cholesterol, NDR = no diabetes retinopathy, SBP = systolic blood pressure, TBIL = total bilirubin, TC = total cholesterol, TG = triglyceride, WBC = white blood cell.

### 3.3. Multiple logistic regression analysis of the relationship between AST/ALT and DR

Multiple logistic regression models were used to analyze the relationship between the AST/ALT ratio and DR. In Model I (adjusted for age and sex), the risk of DR increased by 30% with a 1 standard deviation increase in the AST/ALT ratio (OR = 1.30, 95% CI: 1.13–1.50, *P *< .001). Furthermore, the risk of DR increased by 36% in Model II after adjusting for other covariates (OR = 1.36, 95% CI: 1.16–1.59, *P* < .001). For the sensitivity analysis, we categorized the AST/ALT ratio and processed it according to the third quantile. The results indicated that the AST/ALT ratio was a risk factor for DR. The trend test showed that the risk of DR increased by 47%, with a 1 standard deviation increase in the AST/ALT ratio (OR = 1.47, 95% CI: 1.20–1.80, *P*-trend = 0.014) (Table 3).

**Table 3:** Correlation between AST/ALT ratio and DR by multiple regression equations.

Exposure	Non-adjusted	Adjust I	Adjust II
	OR (95% CI) *P* value	OR (95% CI) *P* value	OR (95% CI) *P* value
AST/ALT *Z* score	1.30 (1.14, 1.49) < .0001	1.30 (1.13, 1.50) .0002	1.36 (1.16,1.59) < .0001
AST/ALT			
Q1 (0.43–0.78)	Ref	Ref	Ref
Q2 (0.78–1.04)	1.42 (0.99, 2.04) .0547	1.41 (0.98, 2.04) .0626	1.75 (1.17, 2.61) .0060
Q3 (1.04–2.26)	1.81 (1.28, 2.57) .0009	1.79 (1.23, 2.60) .0022	2.08 (1.37, 3.18) .0006
*P* for trend	.0001	.0004	.0002

Non-adjusted model adjusted for: none.

Adjust I model adjusted for: sex, age.

Adjust II model adjusted for: sex, age, smoking, alcohol, SBP, DBP, BMI, HBAIC, FPG, 2HPG, TBIL, DBIL, UA, TG, TC, HDL-C, LDL-C, WBC.

**Figure 2.**
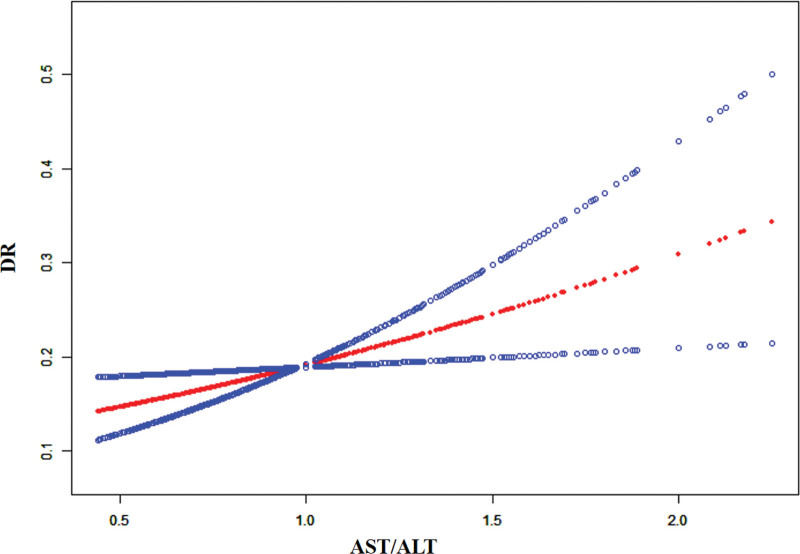
Relationship between AST/ALT ratio with diabetic retinopathy. Adjusted sex; age; SBP; DBP; BMI; HBAIC; FPG; 2HPG; smoking; alcohol; TBIL; DBIL; UA; TG; TC; HDL-C; LDL-C; WBC. AST/ALT = aspartate transaminase/alanine aminotransferase.

As shown in Figure 2, curve fitting was employed to examine the correlation between the AST/ALT ratio and DR, revealing that the risk of DR increased as the AST/ALT ratio increased (*P *= .014).

**Figure 3.**
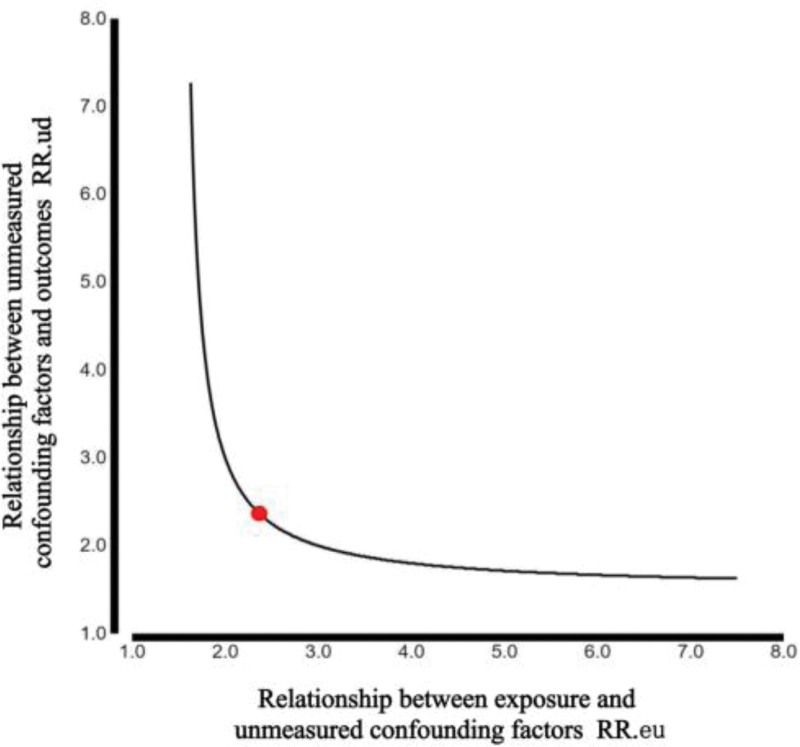
Sensitivity analysis result: *E* value.

We used an *E* value calculator to assess the impact of unmeasured confounding factors. The results indicated an *E* value of 2.37 (Fig. 3).

### 3.4. Correlation between AST/SLT ratio and DR in different subgroups

Hierarchical and interaction analyses illustrated that the relationship between the AST/ALT ratio and DR remained consistent across different subgroups based on age, sex, BMI, and diabetes duration. All results were adjusted for relevant factors, except for stratified variables (Table 4).

**Table 4:** Correlation between AST/ALT ratio and DR in different subgroups.

	N	DR OR (95% CI)	*P* value
Sex			
Male	795	1.18 (0.92, 1.50)	.2103
Female	570	1.43 (1.11, 1.83)	
Age (yrs)			
<50	462	1.30 (0.95, 1.78)	.6201
≥50	903	1.20 (0.98, 1.47)	
DM course (mo)			
≤24	378	1.10 (0.78, 1.56)	0.2629
>24, ≤81	408	1.26 (0.92, 1.73)	
>81	394	1.47 (1.12, 1.94)	
BMI (kg/m^2^)			
≤18.5	10		.6748
>18.5, ≤24	464	1.30 (1.00, 1.69)	
>24	885	1.18 (0.95, 1.48)	
HBAIC (%)			
≤6.5	339	1.41 (1.01, 1.99)	.5342
>6.5	1026	1.22 (1.00, 1.49)	
FPG (mmol/L)			
<7.0	489	1.16 (0.87, 1.55)	.5638
≥7.0	872	1.29 (1.04, 1.61)	
2HPG (mmol/L)			
<11.1	496	1.32 (0.98, 1.77)	.4971
≥11.1	849	1.23 (1.00, 1.52)	

Adjusted: sex, age, SBP, DBP, BMI, HBAIC, FPG, smoking, alcohol, TBIL, DBIL, UA, TG, TC, HDL-C, LDL-C, WBC, 2HPG, DM course, except for hierarchical variables.

2HPG = 2-hour post-load glucose, BMI = body mass index, DM course = course of diabetes, FPG = fasting plasma glucose, HbA1c = glycated haemoglobin.

### 3.5. Mediation analysis

**Figure 4.**
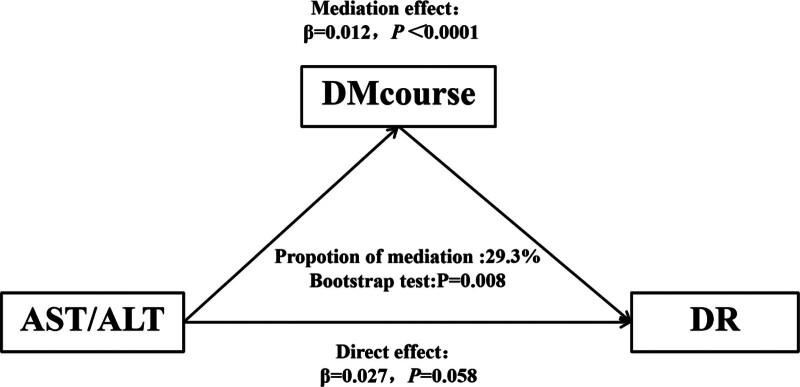
Mediation analysis of the association between AST/ALT ratio and diabetic retinopathy. AST/ALT = aspartate transaminase/alanine aminotransferase; DM course = course of diabetes; DR = diabetic retinopathy.

Diabetes duration emerged as a significant factor in the occurrence of DR. Table 1 indicates an association between a higher AST/ALT ratio and prolonged diabetes duration. A bootstrap test was conducted to evaluate the potential mediation effects in the correlation between the ASL/ALT ratio and DR. As depicted in Figure 4, the proportion of the mediated effects of diabetes duration between the AST/ALT ratio and DR was calculated to be 29.3% (bootstrap test: *P *= .008).

## 4. Discussion

This study aimed to explore the relationship between the AST/ALT ratio and DR. In this cross-sectional study, we report for the first time that the AST/ALT ratio is positively correlated with DR. This indicates that the AST/ALT ratio is a risk factor for DR. Furthermore, after adjusting for relevant factors, this correlation persisted. Therefore, the AST/ALT ratio may be a useful predictor of DR.

The AST/ALT ratio is a common and easily obtained indicator that has attracted the attention of researchers. It was first proposed by researchers in 1957 and is often used to assess liver function and indicate the severity of liver disease.^[1]^ Importantly, the AST/ALT ratio has also emerged as a significant indicator of extracellular diseases, predicting the occurrence and prognosis of nasopharyngeal, rectal, prostate, and other tumors.^[15–17]^ Additionally, it can predict the risk of death in patients with sepsis.^[18]^ It is also an effective indicator of cardiovascular diseases. An elevated AST/ALT ratio is a risk factor for cardiovascular and all-cause mortality in patients with acute myocardial infarction and hypertension.^[19–21]^

It has been demonstrated that an increase in the AST/ALT ratio not only indicates liver function impairment but may also reflect metabolic disorders in the entire body.^[21]^ The occurrence and development of DR are complex processes that involve at least 9 types of retinal cells.^[22]^ Furthermore, DR is caused by the dysfunction of retinal neurovascular units, including cell loss, production of inflammatory factors, destruction of new blood vessels, and the blood–retinal barrier.^[23]^ Studies have reported that dyslipidemia, mitochondrial apoptosis, and oxidative stress may be the main pathological changes in DR.^[24–27]^ Previous studies have shown that an increase in the AST/ALT ratio is indicative of oxidative stress, which is one of the reasons why AST/ALT is a risk factor for DR. Additionally, the AST/ALT ratio can also be used as an indicator to assess the risk of arteriosclerosis.^[28,29]^ Atherosclerosis is a perilous factor for microvascular complications of diabetes, including retinopathy, which is well-known to researchers.^[30–32]^ Though the specific mechanism is unknown, it may involve endothelial cell dysfunction, chronic inflammation, and increased oxidative stress.^[33,34]^ In addition, it is well known that insulin resistance is a risk factor for DR^[35]^ and that the AST/ALT ratio is a marker of insulin resistance.^[36]^ This study also found that SBP and FPG were risk factors for DR, which is consistent with the results of previous studies.^[37,38]^

Moreover, the course of diabetes is a risk factor for all complications including DR, and the results of this study confirm this viewpoint. Notably, the AST/ALT ratio increases with age.^[37]^ We found that among patients with type 2 diabetes, those with higher AST/ALT ratios tended to be older and had a longer duration of diabetes. Mediation analysis was used to evaluate whether the duration of diabetes played a mediating role in the occurrence of AST/ALT ratio and DR. We found that 29.3% of the risk of AST/ALT ratio increasing DR was mediated by the duration of diabetes. To evaluate unmeasured confounding factors, we calculated *E* value.^[13]^ The results showed that the *E* value was >2, indicating that it was unlikely that there were unmeasured confounding factors affecting the stability of the results. Recently, some researchers found that an increase in the AST/ALT ratio may lead to diabetic nephropathy by increasing the level of inflammatory cytokines, suggesting that it is an independent risk factor for diabetic nephropathy (DN).^[39]^ It is well-known that both DR and DN are microvascular complications of diabetes and that they may have similar pathogenesis. In conclusion, we speculate that a high AST/ALT ratio is an independent risk factor for DR. The results support our conjecture in this study, and the results remained stable after adjusting for the relevant variables. To the best of our knowledge, this is the first report on the correlation between AST/ALT ratio and DR, and the stability of the results was verified through hierarchical and interaction analyses. Mediation analysis and *E* values were the strengths of this study.

This study had several limitations. First, this was a single-center cross-sectional study, and the causal relationship between AST/ALT levels and DR could not be determined, necessitating further investigation through longitudinal research. Second, the number of patients with DR in this study was relatively small and there was no further stratification based on severity. Third, the collection of indicators related to oxidative stress could have enhanced the persuasiveness of the data. Finally, although we employed different analytical methods to support our findings, caution is needed when interpreting these results as some observed associations may be coincidental. Therefore, additional real-world data were required for validation.

## 5. Conclusion

Our research has shown a significant positive correlation between the prevalence of DR and an elevated AST/ALT ratio, highlighting it as an independent risk factor for DR. This AST/ALT ratio, owing to its ease of accessibility, has the potential to serve as a valuable predictor for the onset of DR.

## Acknowledgments

We would like to thank Editage (www.editage.cn) for the English language editing.

## Author contributions

**Conceptualization:** Jian Luo, Shenglian Gan.

**Methodology:** Jian Luo, Quan Zhou.

**Resources:** Jian Luo.

**Validation:** Jian Luo, Fang Yu.

**Writing – original draft:** Jian Luo, Xueyan Wu.

**Writing – review & editing:** Jian Luo, Shenglian Gan.

**Supervision:** Fang Yu.

**Data curation:** Haifeng Zhou, Qin Liu.

**Visualization:** Haifeng Zhou.

**Investigation:** Xueyan Wu, Qin Liu.

**Formal analysis:** Quan Zhou.

**Software:** Quan Zhou.

**Project administration:** Shenglian Gan.
